# Leptomeningeal Disease: Current Approaches and Future Directions

**DOI:** 10.1007/s11910-025-01412-y

**Published:** 2025-03-18

**Authors:** Ugur Sener, Jessica A. Wilcox, Adrienne A. Boire

**Affiliations:** 1 Department of Neurology, Mayo Clinic, Rochester, MN USA; 2 Department of Medical Oncology, Mayo Clinic, Rochester, MN USA; 3 Department of Neurology, Memorial Sloan Kettering Cancer Center, New York, NY USA; 4 Brain Tumor Center, Human Oncology and Pathogenesis Program, Memorial Sloan Kettering Cancer Center, New York, NY USA

**Keywords:** Leptomeningeal disease, Leptomeningeal metastases, Craniospinal radiation, Intrathecal therapy, Central nervous system metastases, Cerebrospinal fluid

## Abstract

**Purpose of Review:**

Leptomeningeal disease (LMD), or spread of cancer cells into the pia and arachnoid membranes encasing the brain and spinal cord, is associated with high symptom burden and poor survival at 2 to 5 months. Conventional treatments including photon-based radiation therapy, systemic chemotherapy, and intrathecal chemotherapy demonstrate limited efficacy. Despite significant successes for a range of solid tumors, immunotherapy has not yet demonstrated significant efficacy in management of LMD. Advances in understanding of LMD pathophysiology, improved diagnostics, and novel therapeutics are shifting this paradigm. In this article, we review diagnostic and treatment challenges associated with LMD.

**Recent Findings:**

We discuss the use of novel cerebrospinal fluid (CSF) analysis techniques such as circulating tumor cell and CSF cell-free DNA assessment to overcome limitations of conventional diagnostic modalities. We then review advances in treatment including clinical trial data demonstrating efficacy of proton craniospinal radiation to treat the entire neuroaxis. We discuss emerging data regarding targeted therapeutics conferring durable survival benefit.

**Summary:**

Novel therapeutics and combinatorial treatment approaches will likely further improve outcomes for patients with LMD.

## Introduction

Leptomeningeal disease (LMD) describes spread of cancer cells into the cerebrospinal fluid (CSF)-filled membranes encasing the brain and spinal cord [1, 2]. LMD describes a particularly destructive pattern of cancer progression associated with median overall survival (mOS) of 2 to 5 months [3]. Reflecting the diffuse nature of disease location, LMD is associated with significant symptom burden including headache, seizures, cranial neuropathies, weakness, and gait difficulty [4]. LMD related to solid tumors most commonly occurs in the setting of breast cancer, lung cancer, and melanoma; though in practice, any tumor may result in LMD [1, 5]. LMD has been reported at initial cancer diagnosis or as part of initial intracranial involvement in 2–12% of cases, but more often occurs as a late complication [6]. Prognosis of LMD varies by primary tumor type and tumor molecular markers [2]. For example, LMD related to breast cancer is generally associated with superior survival than that observed with lung cancers [5–8]. Within breast cancer subtypes, LMD related to human epidermal growth factor receptor 2 (HER2) and hormone receptor positive breast cancer carries a better prognosis compared to triple negative tumors [7]. Treatment approaches for LMD include radiation therapy, molecularly targeted systemic therapy in applicable cases, immunotherapy, systemic chemotherapy, and intrathecal chemotherapy [9]. In this review we will discuss recent advances in pathophysiology, diagnosis, and treatment of LMD.

## Pathophysiology of Leptomeningeal Disease

The leptomeninges consist of the pia and arachnoid membranes. The arachnoid is laminated to the dural surface, forming the outer limit of the central nervous system. The pia adhere to the cortical surface. Interaction between the pia and astrocytic endfeet leads to generation of extracellular matrix forming the glia limitans. Circulating CSF, generated by the choroid plexuses (and, to a lesser extent, the ependyma) fills the ventricular system and the space between these two membranes. Once cancer cells gain access to this space and successfully grow within the space, LMD results [9]. Mechanisms of tumor cell entry into the CSF include hematogenous dissemination through the choroid plexus or retrograde venous extension [10, 11]. Tumor cells may also directly seed the CSF through parenchymal brain or spinal cord metastases [11]. Upregulation of complement component 3 (C3) has been identified as a critical mechanism through which cancer cells activate the C3a receptor in the choroid plexus, thereby disrupting the blood-CSF barrier [11]. Although C3a is necessary for cancer cell entry through the barrier, it is not sufficient to mediate entry of tumor cells into the space. Mechanism(s) governing cancer cell entry into the leptomeninges represent an active area of basic research.

Within the leptomeningeal space, LMD generates two phenotypes [12]. Tumor cells may adhere to the leptomeninges, creating a plaque-like appearance that can be detected on magnetic resonance imaging (MRI) of the neuroaxis. Alternatively, tumor cells may be free-floating, detected by CSF cytology. LMD related to free-floating tumor cells has been associated with poorer survival compared to plaque-like adherent LMD in the setting of breast and lung cancer [12]. Similarly, in a prior retrospective study of 225 patients with LMD, cytology positive for tumor cells was noted as a negative prognostic factor [3]. In the same study, among patients with cytology negative for tumor cells, presence of nodular disease on MRI was a negative prognostic factor compared to radiographic linear enhancement on MRI [3].

Tumor cells in the leptomeninges are faced with an unfavorable environment with profound nutritional scarcity. One mechanism that has been identified to facilitate cancer cell survival within the space is through expression of iron-scavenging protein and receptor pair, lipocalin-2 (LCN2) and SCL22A17 [13]. Cancer cells secrete LCN2 into the CSF to sequester iron from CSF macrophage, thereby impairing macrophage function and supporting metastatic growth [13]. Following promising results from disruption of this pathway in preclinical models, intrathecal administration of the iron chelator deferoxamine for treatment of LMD is currently under investigation (NCT05184816) [14].

Further mechanistic understanding of how tumor cells survive and proliferate within the CSF while avoiding the immune system is likely to yield additional vulnerabilities that can be exploited by novel therapeutics.

## Diagnostic Advances in Leptomeningeal Disease

**Fig. 1:**
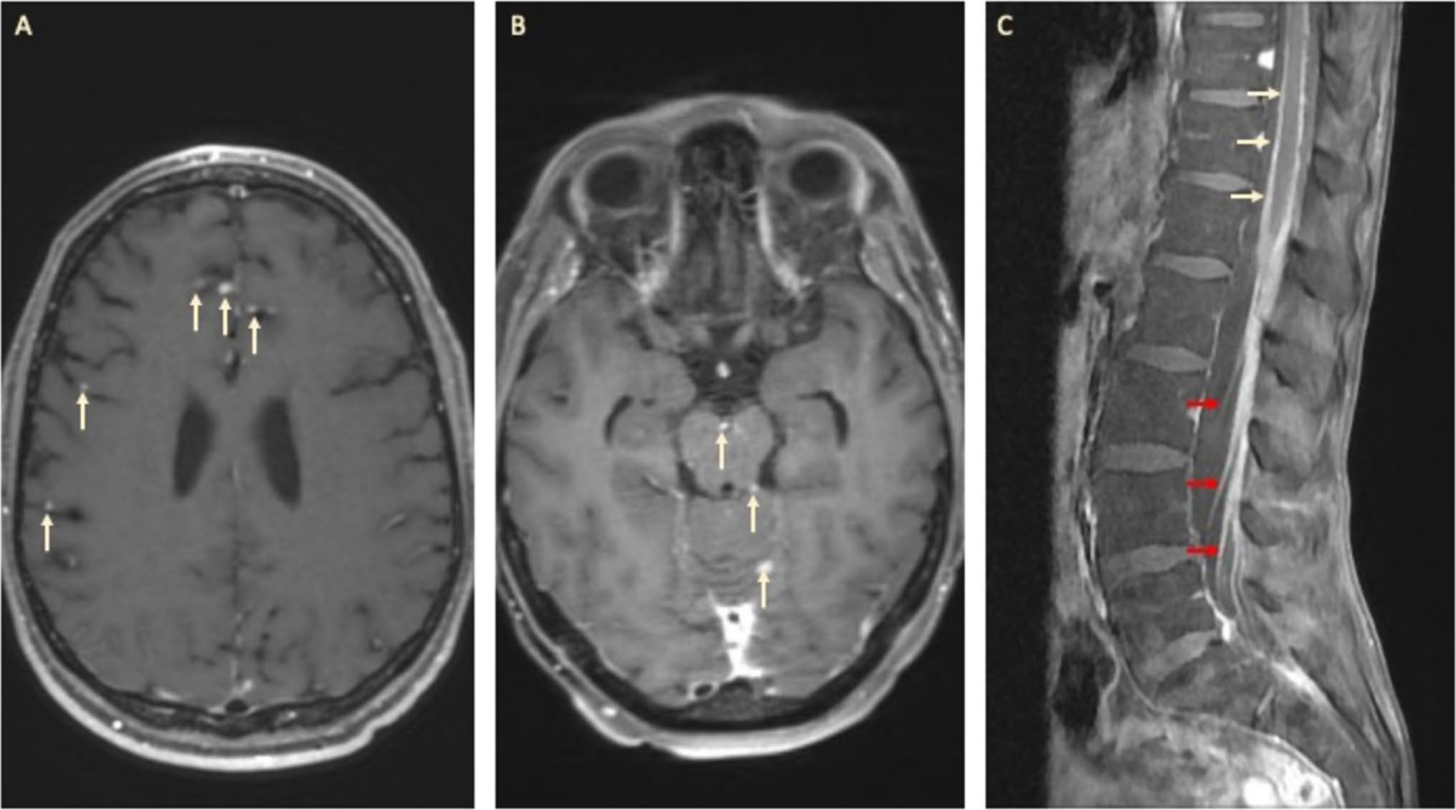
Intracranial and Spinal Appearance of Leptomeningeal Disease**. A** MRI brain with contrast demonstrating multiple leptomeningeal deposits (arrows). **B** MRI brain with contrast demonstrating leptomeningeal deposits involving the brainstem and cerebellum (arrows). **C** MRI of the lumbar spine with contrast demonstrating diffuse enhancement along the spinal cord (yellow arrows) and cauda equina nerve roots (red arrows)

Because LMD encompasses the entire leptomeningeal space, diagnostic and monitoring evaluation of LMD requires imaging of the entire neuroaxis as well as CSF analysis [9]. On conventional MRI, intracranial LMD can appear as contrast enhancement along cranial nerves, surrounding the brainstem, and within the cerebellar folia (Fig. 1) [15]. Along the spinal cord, LMD can appear as enhancement over the cord surface or as clumping and enhancement of the cauda equina or individual nerve roots [15]. However, radiographic findings related to LMD can be subtle, and the differential diagnosis for leptomeningeal enhancement is broad. Moreover, a normal MRI of the neuroaxis does not exclude the diagnosis [2].

Radiographic assessment of LMD also represents a challenge for determination of disease progression and response to treatment. The Response Assessment in Neuro-Oncology (RANO) criteria for LMD have been proposed to address this issue and improve radiographic disease assessment [16]. However, these criteria have not yet been prospectively validated in their entirety. Perhaps due to the complexity of these criteria, their use in routine clinical practice has been limited [17]. More recently, a new imaging scorecard was validated in a small cohort of 20 patients [18]. Joint guidelines from the European Association of Neuro-Oncology (EANO) and European Society for Medical Oncology (ESMO) are also available for follow up and disease assessment [3, 19, 20].

The CSF biochemical and cytological profile is abnormal in the setting of LMD, with lymphocytic pleocytosis, elevated protein, and decreased glucose representing the most common findings [21]. Conventional cytology allows for direct visualization of tumor cells within CSF and is the gold standard for diagnosis of LMD. However, yield from a single CSF sample is low and the false negative rate for conventional cytology is high [15]. While sensitivity of CSF cytology is improved with repeated sampling, this can be cumbersome for patients and delay diagnosis [22].

Novel approaches for CSF evaluation include CSF circulating tumor cell (CTC) and cell-free tumor DNA (ctDNA) assessment [2]. CTCs can be detected based on surface protein expression. As an example, epithelial cell adhesion molecule (EpCAM) is expressed on the surface of epithelial cells, including tumor cells of breast or lung cancer origin [23–25]. Thus, EpCAM-based assays can be used for detection of CTCs in the CSF. Similarly, High-Molecular Weight-Melanoma-Associated Antigen/Melanoma-associated Chondroitin Sulfate Proteoglycan (HMW-MAA/MCSP) has been explored for detection of CTCs related to melanoma [26]. In a prospective study of patients with breast cancer, CSF CTC detection demonstrated superior sensitivity in comparison to traditional cytology to detect LMD [27]. Similar findings were reported in lung cancer LMD; CTC sensitivity was 94%, cytology 76% [28].

In addition to commercially available cell-surface protein-based assays, flow cytometry can also establish the diagnosis of LMD. In this technique, fluorescently labeled antibodies against cell surface proteins of interest detect of CSF CTCs [29]. Use of flow cytometry for detection of LMD from solid tumors remains extremely limited; surface antigens are less established in these tumors. However, in hematologic malignancies, this technique is standard-of-care [30].

CSF CTC measurement also provides a quantitative measure of LMD burden with potential clinical utility for treatment response assessment and detection of disease progression. As an example, in 15 patients with LMD related to breast cancer, higher CSF CTC enumeration was associated with more rapid disease progression [31]. Importantly, high CSF CTC burden did not necessarily correlate with more pronounced radiographic appearance of LMD, underscoring the ability of this quantitative biomarker assessment to provide diagnostic, prognostic, and response assessment information [31]. In a subsequent retrospective study, increased CSF CTC count was associated with increased risk of mortality as a continuous variable. Greater than or equal to 61 CSF-CTCs per 3 ml of CSF was identified as the optimal cutoff, at which risk of mortality doubled [32]. However, limitations of CSF CTC quantification include limited availability of commercial assays at most centers, possibility of epithelial-to-mesenchymal transformation theoretically reducing the yield of EpCAM-based assays, and need to develop assays or flow cytometry techniques for surface proteins associated with different malignancies [2].

Tumor cells within the CSF shed DNA, which can be detected by CSF ctDNA analysis [33]. As an example, in a prior study of 43 CSF samples from 22 patients with LMD, cytology was positive for malignant cells in 72% of the samples, compared to 93% positive for CSF ctDNA [34]. CSF ctDNA has successfully been employed for diagnosis and classification of primary CNS tumors [35, 36]. The quantitative nature of CSF ctDNA may have utility in response assessment and prognostication [37]. However, the qualitative and descriptive aspects of CSF ctDNA analysis are of perhaps the greatest value, enabling identification of targetable mutations with potentially actionable targeted therapies or identifying divergent mutational profiles between CNS and the primary tumor site [33]. For instance, a study of patients with lung cancer harboring epithelial growth factor (EGFR) mutations revealed unique mutations and copy number variations in CSF ctDNA not detected in analysis of the primary tumor [38]. In a more recent study, analysis of CSF ctDNA from patients with LMD secondary to lung cancer captured molecular characteristics and heterogeneity of metastatic disease [39]. Thus, examination of CSF ctDNA may identify resistance mechanisms and suggest therapeutic strategies unique to the CNS compartment [2]. Limitations to the use of CSF ctDNA include lack of standardized processes for DNA extraction, limited availability of the testing for widespread use and the processing time required that may preclude clinical decision-making based on results [33]. Furthermore, ctDNA may be shed into the CSF from both parenchymal brain metastases and LMD. Thus, the presence of CSF ctDNA does not provide diagnostic certainty. Optimized protocols for CSF ctDNA isolation are under development and clinical utility of this test for molecular characterization of LMD is likely to increase [40].

Further advances in advanced diagnostic tools for detection and monitoring of LMD may in time permit earlier detection, improved diagnostic certainty, and more widely available quantitative response assessment. Utilization of CSF next-generation sequencing has the potential to identify resistance mechanisms, informing development of new therapies. Table 1 summarizes emerging CSF diagnostic strategies for LMD.

**Table 1:** Emerging Cerebrospinal Fluid Analytic Strategies for Leptomeningeal Disease

	CTC Analysis	ctDNA Analysis
Technique	Tumor cell detection based on expression of surface proteins	Sequencing of DNA released from tumor cells into the CSF
Advantages	• Improved sensitivity compared to conventional cytology • Quantitative assessment allows longitudinal monitoring of therapeutic response • Potential prognostic value based on quantity of CTCs	• Potential to detect targetable tumor genetic alterations • Potential to detect mutational divergence between CNS and extracranial tumor cells
Disadvantages	• Limited availability • Requires specialized equipment • Individual assays specific to individual tumor types	• Poor specificity: May also originate from parenchymal brain metastases, limiting diagnostic attribution • Limited availability • Lack of standardization • Prolonged processing time

## Therapeutic Advances in Leptomeningeal Disease

Joint EANO and ESMO guidelines provide guidance for management based on tumor type, presence of brain metastases, and extent of systemic disease [19, 20]. Treatment of LMD primarily involves radiation therapy to the symptomatic sites followed by CNS-penetrant systemic therapy. Due to the diffuse nature of LMD, surgical resection is not possible. In select cases, ventriculoperitoneal shunting (VPS) can be considered for palliation of symptoms related to obstructive hydrocephalus [41]. In a study of 190 patients with LMD who underwent CSF diversion, symptomatic relief was noted among 83% [42]. Complications were uncommon with 5% of patients experiencing a shunt related infection and 6.3% having a subdural hygroma/hematoma [42]. Peritoneal carcinomatosis secondary to VPS is exceedingly rare [43]. Recently, combination of an occludable VPS with IT chemotherapy was retrospectively associated with better survival compared to occludable shunt alone [44].

### Radiation Therapy

Historically, LMD has been treated with photon-based whole brain radiation therapy (WBRT) and focal spinal radiation therapy (RT) to symptomatic sites of disease [45]. The disadvantage of this technique is that it does not address disease within the entire CSF compartment, and therefore has not been associated with a survival benefit. Photon-based craniospinal irradiation (CSI) is an option, but this is associated with profound toxicities in adults and is not recommended [46].

Proton CSI has emerged as a more tolerable alternative to photon CSI to treat the entire neuroaxis [47]. Rather than the progressive dose fall-off seen with photon radiation, protons deposit most of their energy within a few millimeters of their end range, thereby eliminating exit radiation dose and limiting off-target toxicities [48]. The resultant efficient and focused delivery of RT to the craniospinal axis largely spares the vertebral bone marrow, reducing risk of myelotoxicity. Anterior torso organ systems receive less radiation with proton compared to photon CSI, resulting in less toxicity to these organs.

Safety and tolerability of proton CSI for patients with LMD from solid tumors was established in a phase I clinical trial [47]. In this study, among 24 patients with LMD treated with proton CSI, median central nervous system (CNS) progression free survival (PFS) was 7 months and mOS was 8 months [47]. The most common toxicities were fatigue and self-limiting lymphopenia and thrombocytopenia.

In a subsequent clinical trial, proton CSI was compared directly to involved field photon radiotherapy (IFRT) [49]. IFRT was defined as WBRT and/or focal spinal RT to symptomatic areas. Forty-two patients treated with proton CSI were compared to 21 patients treated with IFRT. A significant benefit in CNS PFS was observed with proton CSI (median 7.5 months; 95% CI, 6.6 months to not reached) compared with IFRT (2.3 months; 95% CI, 1.2 to 5.8 months; P, 0.001). OS for proton CSI was 9.9 months (95% CI, 7.5 months to not reached) compared to 6.0 months for IFRT (95% CI, 3.9 months to not reached; P 5 0.029).

Importantly, the proton CSI trials enrolled a heterogeneous population of patients, some with actionable tumor genetic mutations. In practice, toxicities (particularly high-grade fatigue) are most prominent in patients greater than 60 years of age, or those that are heavily pretreated. Further, limited availability of centers capable of delivering proton therapy limits generalizability of the findings. Nevertheless, the results of the phase 1 and phase 2 trials are promising and represent a significant advancement in treatment of LMD. Proton CSI should be considered for select patients with LMD when possible, particularly for those in whom the goal of treatment is durable disease control. Sequencing CNS-active systemic therapy with proton CSI may further improve patient outcomes [50, 51].

### Conventional Systemic Therapies

LMD is often encountered in the setting of progressive extracranial disease [20]. As such, systemic therapies that can target both extracranial and CNS compartments represent an attractive option. However, systemically-administered, cytotoxic chemotherapies have demonstrated limited efficacy in treatment of LMD. High-dose IV methotrexate is among the most frequently employed approaches but is associated with a modest survival benefit [52]. Despite frequent use in other CNS tumors, single agent temozolomide has not demonstrated efficacy for LMD related to solid tumors [53]. Capecitabine alone or in combination with trastuzumab represents one tumor-specific systemic therapy option with modest benefit in treatment of LMD related to breast and esophageal cancer [54, 55].

### Intrathecal Therapy

Intrathecal (IT) chemotherapy describes direct administration of chemotherapy into the CSF space by a lumbar puncture or through the use of an intraventricular catheter (e.g., Ommaya reservoir) [2]. To safely receive IT chemotherapy, patients must have non-bulky disease with no CSF flow obstruction [21]. In a retrospective study, intraventricular therapy via Ommaya catheter was associated with better OS compared to IT chemotherapy administered into the lumbar cistern [56].

Methotrexate, cytarabine, topotecan, pemetrexed, and thiotepa represent the most commonly administered IT chemotherapy agents [2]. The mOS from early studies of IT chemotherapy range from 2–4 months. However, comparative clinical trials are lacking, optimal dosing regimens are unknown, and the patient population from these historical trials do not reflect the modern era of cancer-directed therapies [57–61]. More modern trials of IT chemotherapies are suggestive of better survival outcomes [62–65]. A common pattern of IT chemotherapy administration in clinical practice involves use of methotrexate, cytarabine, topotecan, or thiotepa twice weekly for four weeks [66]. If cytologic response is favorable following the initial four-week period, treatment can continue once weekly for four weeks and then once monthly for maintenance.

IT chemotherapy can be associated with toxicities related to chemical meningitis including fever, headaches, and nausea [66]. IT methotrexate is associated with leukoencephalopathy in certain cases, particularly in the post-RT setting [67]. Very limited data is available for combinatorial IT chemotherapy strategies, such as a small study of cytarabine and methotrexate combination demonstrating improved disease control compared to methotrexate alone [68]. In another study investigating combinatorial therapy, IT liposomal cytarabine combined with systemic chemotherapy was associated with improved survival compared to systemic chemotherapy alone among patients with LMD related to breast cancer [62]. On the other hand, a study of IT methotrexate or cytarabine combined with systemic chemotherapy compared to systemic chemotherapy demonstrated no added benefit from IT chemotherapy among patients with LMD related to breast cancer [61].

IT administration of immunotherapy is a consideration, but published experience remains limited to small studies and case reports. In a retrospective report of 43 patients with LMD from melanoma treated with IT interleukin-2 (IL-2), mOS was 7.8 months but was accompanied by a high rate of symptomatic intracranial hypertension [69]. A subset of patients achieved long-term disease control with 5-year OS rate reported at 13%. These findings suggest a subset of patients with LMD secondary to melanoma may derive significant benefit from IT IL-2, but patient or tumor-specific factors contributing to this prolonged survival remain unclear. Combination of IT immune checkpoint inhibitor (ICI) therapy with intravenous ICI is an emerging treatment option for ICI-sensitive cancers [70]. Interim results from a phase 1 study of IT plus IV nivolumab in patients with melanoma LMD demonstrated a preliminary mOS of 4.9 months with tolerable safety data [70, 71].

Similarly, IT administration of molecularly targeted therapies may be a consideration in select settings. Administration of IT trastuzumab was evaluated in a clinical trial for patients with LMD related to HER2-positive breast cancer [72]. Thirty-four patients with LMD were treated. During the phase 1 portion of the study, mOS for patients treated with the 80 mg dose selected for phase 2 was 8.3 months; mOS during the phase 2 portion of the study was 10.5 months [72]. In another study, 7 patients with HER2-positive breast cancer received IT trastuzumab [73]. mOS was 13.7 months in the IT trastuzumab group, compared to 9.3 months among patients who did not receive IT therapy [73]. IT trastuzumab in the setting of HER2 positive esophageal carcinoma was also evaluated, but data is very limited with one patient experiencing durable disease control and the other experiencing rapid progression and short survival [74]. While these findings were encouraging, a meta-analysis of 45 publications representing 208 patients identified no difference in mOS between IT trastuzumab compared to oral or IV administration of HER2 targeted therapy [75].

Based on current evidence, use of conventional IT therapy for LMD is best reserved for patients with reasonable performance status, limited CNS-penetrant systemic therapy options, reasonably controlled extracranial disease, and/or in the context of a clinical trial. The potential utility of IT-based immunotherapy for melanoma and HER2 targeted therapy for LMD related to HER2-positive cancer is additionally encouraging. Further studies may better delineate a role for IT therapy for selected subpopulations of patients based on patient and tumor characteristics.

### Immunotherapy

Checkpoint regulators such as programmed cell death protein 1 (PD-1) downregulate T cell activation [76]. ICIs such as the anti-PD1 agent pembrolizumab work by blocking these checkpoints with intent to generate a sustained anti-tumor immune response. ICIs have proven efficacy in numerous systemic malignancies including lung cancer, breast cancer, and melanoma [77]. Despite these successes, the role of ICIs for LMD remain limited.

Two clinical trials have evaluated pembrolizumab in mixed tumor populations of LMD [78, 79]. In the first study, 13 patients with LMD were treated with pembrolizumab with median OS at 4.9 months [78]. In the second study, 20 patients with LMD were treated with pembrolizumab giving an OS of 3.6 months, for a three-month OS of 60% [79].

In a study of melanoma patients with brain metastases, four patients with LMD were treated with anti-PD1 ICI nivolumab monotherapy [80]. No patients responded to treatment. In another study combining anti-cytotoxic T-lymphocyte-associated protein 4 (CTLA-4) ICI ipilimumab and nivolumab for 18 patients with LMD, median OS was 2.9 months [81]. Similar results were noted in a retrospective review of ICIs for LMD related to lung cancer with mOS reported at 3.7 months [82]. In a subsequent systematic review of 61 patients with LMD related to solid tumors across 14 studies, mOS was 6.3 months with patients receiving no corticosteroids experiencing longer survival [83].

Taken together, these mOS data from small studies with ICI monotherapy for management of LMD are comparable to mOS associated with conventional treatments such as systemic or intrathecal chemotherapy. To date, ICIs have not satisfactorily improved survival from LMD as single agents. Combinatorial strategies such as use of ICIs with proton CSI, use of dual ICI, or combinations of other systemic and IT therapies with ICIs are currently under investigation.

### Molecularly Derived Targeted Therapies

Systemic therapies targeting driver mutations have remarkable efficacy in management of a range of malignancies. As an example, EGFR and anaplastic lymphoma kinase (ALK) targeted therapies have dramatically altered management of non-small cell lung cancer (NSCLC). V-raf murine sarcoma viral oncogene homolog B1 (BRAF) and mitogen-activated protein kinase (MEK) inhibitors are used in treatment of melanoma and other BRAF-mutant tumors. These agents have also demonstrated efficacy in LMD related to solid tumors.

EGFR-targeted tyrosine kinase inhibitor (TKI) osimertinib has demonstrated significant efficacy in treatment of LMD related to EGFR-mutant NSCLC [84–86]. In the phase 1 BLOOM study, 41 patients with LMD secondary to EGFR-mutant NSCLC were treated with osimertinib with an mOS of 11 months [84]. Similarly, within the AURA program, 22 patients with LMD secondary to EGFR-mutant NSCLC were treated with osimertinib and the mOS was 11.1 months [85]. In a recent meta-analysis of 243 patients and 282 lines of EGFR-TKI for patients with LMD from NSCLC, median OS was 14.5 months [86]. Osimertinib was associated with improved outcomes compared to other EGFR TKIs [86].

While early-generation ALK inhibitor crizotinib had limited CNS activity, the dual ALK and ROS1 inhibitor lorlatinib has been associated with CNS responses ranging from 8 to 22 months [87]. Among 11 patients with LMD due to NSCLC with ALK (*n* = 9) or ROS1 (*n* = 2) mutations, lorlatinib therapy demonstrated an intracranial objective response rate (ORR) of 45%; survival data was not provided [88]. Responses to the ALK inhibitor alectinib have also been reported in setting of LMD secondary to NSLC [89, 90].

Responses to BRAF/MEK inhibitor combinations dabrafenib/trametinib and encorafenib/binimetinib have been reported [91–93]. While individual case reports have indicated some instances of durable responses, patients often experience progression on these targeted therapeutics at the time of LMD diagnosis, limiting their clinical utility specifically for management of LMD due to BRAF-mutant tumors [2].

For patients with LMD related to HER2-positive breast cancer, the antibody drug conjugate trastuzumab deruxtecan has been associated with durable responses despite uncertain CSF penetration [94, 95]. In a retrospective study of 8 patients with LMD secondary to HER2-positive breast cancer, mOS was 10.4 months [95]. In the ROSET-BM study, among 19 patients with radiographic LMD, 12-month overall survival rate was 87.1% though cytologic-positivity and responses were not reported [94]. The selective HER2 inhibitor tucatinib in combination with trastuzumab and capecitabine was associated with improved CNS disease control and survival (CNS-PFS = 9.9 mo; mOS = 18.1 mo) compared to trastuzumab and capecitabine without tucatinib (CNS-PFS = 4.2 mo; mOS = 12.0 mo). While large-scale data is lacking, a PFS of 7 months and OS of 10 months was reported for a patient with LMD secondary to HER2 positive breast cancer treated with tucatinib [96]. Furthermore, preliminary results of a phase 2 study of tucatinib, capecitabine, and trastuzumab in newly diagnosed LMD suggest an LM-ORR of 38% and 100% of patients achieving clinical benefit [97]. Tucatinib, like other small molecule inhibitors, has documented CSF penetration [98],

Another potential therapeutic approach may be trastuzumab deruxtecan, an antibody drug conjugate consisting of anti-HER2 antibody trastuzumab linked to the topoisomerase I inhibitor deruxtecan. Among 19 patients with breast cancer related LMD, trastuzumab-deruxtecan was associated with 12-month survival rate of 87.1%, though CSF cytology data was not reported in this study and clinical experience with the antibody–drug conjugate is so far lacking [94]. It remains to be determined whether combined administration of IT trastuzumab with systemic HER2-acting agents such as tucatinib or trastuzumab-deruxtecan is feasible or associated with any additional benefit.

The vascular endothelial growth factor (VEGF) inhibitor bevacizumab is frequently used in treatment of primary CNS tumors as a steroid-sparing agent for management of symptomatic cerebral edema, even though it is not associated with an OS benefit [99]. Among patients with LMD, high levels of VEGF have been negatively correlated with survival [100]. In a prior study, combination of bevacizumab, etoposide, and cisplatin was associated with CNS response and improved OS in setting of LMD related to breast cancer [101]. Given withdrawal of bevacizumab approval for breast cancer, this combination is not frequently used in the clinic [102]. Similarly, possible benefit from combination of erlotinib with bevacizumab was reported for LMD secondary to EGFR-mutant NSCLC [103]. However, given subsequent development of and clear benefit associated with CNS-penetrant EGFR inhibitor osimertinib, this combination is unlikely to be used clinically.

Clinical experience with molecularly targeted therapies to date indicates clear benefits from CNS-penetrant agents such as osimertinib, lorlatinib, tucatinib, and trastuzumab deruxtecan. Many patients with primary tumors harboring actionable mutations will have already received targeted therapy at the time of LMD diagnosis. Nevertheless, for patients who present with LMD at the time of initial tumor diagnosis or treated with an earlier generation TKI and experiencing progression, targeted therapies represent viable treatment options.

## Conclusion

LMD remains a challenging complication of malignancy resulting in significant morbidity and mortality. Significant advances have been made in diagnosis and treatment of LMD over the past decade, suggesting potential for change. Emerging diagnostic tools such as CTCs and CSF ctDNA provide earlier diagnosis, prior to the onset of irreversible neurological damage. These tools also provide novel ways to monitor the course of LMD, permitting earlier detection of treatment response and guiding personalized treatment based on tumor molecular profiles.

Advances in radiation therapy, notably proton CSI, provide a treatment option for the entire CSF compartment for select patients. Combination proton CSI with systemic therapy may further improve outcomes and is an area of active investigation. Available clinical trial data has demonstrated the potential of CNS-penetrant targeted therapies in LMD. With improved understanding of tumor growth mechanisms and LMD pathophysiology, further systemic therapy options are likely to be developed.

Given the wealth of treatment resistance mechanisms already established in solid tumor malignancies, regardless of primary or site of metastasis, it is unlikely that any single treatment modality will sufficiently treat LMD and achieve long-term survival. Combinatorial strategies individualized to individual tumor profiles and adjusted over the disease course have the highest likelihood to improve local disease control, symptom burden, and survival.

## Key References

- Lamba N, Cagney DN, Catalano PJ, Elhalawani H, Haas-Kogan DA, Wen PY, Wagle N, Lin NU, Aizer AA, Tanguturi S (2023) Incidence proportion and prognosis of leptomeningeal disease among patients with breast vs. non-breast primaries. Neuro Oncol 25 (5):973–983. 10.1093/neuonc/noac249
  - ○ This large single institution review represents a modern epidemiologic study of LMD, highlighting the high incidence of LMD arising from breast cancer and superior survival in patients with breast LMD as opposed to LMD from other solid cancers.
- Chi Y, Remsik J, Kiseliovas V, Derderian C, Sener U, Alghader M, Saadeh F, Nikishina K, Bale T, Iacobuzio-Donahue C, Thomas T, Pe'er D, Mazutis L, Boire A (2020) Cancer cells deploy lipocalin-2 to collect limiting iron in leptomeningeal metastasis. Science 369 (6501):276–282. 10.1126/science.aaz2193
  - ○ This publication highlights an iron-scavenging pathway critical for leptomeningeal cell surivival in the nutrient-sparse CSF. Understanding the unique mechanisms of cancer dissemination and survival within the leptomeningeal space is crucial for the development of novel therapeutics against LMD.
- Le Rhun E, Devos P, Winklhofer S, Lmalem H, Brandsma D, Kumthekar P, Castellano A, Compter A, Dhermain F, Franceschi E, Forsyth P, Furtner J, Galldiks N, Gallego Perez-Larraya J, Gempt J, Hattingen E, Hempel JM, Lukacova S, Minniti G, O'Brien B, Postma TJ, Roth P, Ruda R, Schaefer N, Schmidt NO, Snijders TJ, Thust S, van den Bent M, van der Hoorn A, Vogin G, Smits M, Tonn JC, Jaeckle KA, Preusser M, Glantz M, Wen PY, Bendszus M, Weller M (2022) Prospective validation of a new imaging scorecard to assess leptomeningeal metastasis: A joint EORTC BTG and RANO effort. Neuro Oncol 24 (10):1726–1735. 10.1093/neuonc/noac043
  - ○ This publication outlines the validation of an updated RANO-LM imaging scorecard and represents an international collaboration between the EORTC BTG and RANO to improve response monitoring in LMD.
- Yang JT, Wijetunga NA, Pentsova E, Wolden S, Young RJ, Correa D, Zhang Z, Zheng J, Steckler A, Bucwinska W, Bernstein A, Betof Warner A, Yu H, Kris MG, Seidman AD, Wilcox JA, Malani R, Lin A, DeAngelis LM, Lee NY, Powell SN, Boire A (2022) Randomized Phase II Trial of Proton Craniospinal Irradiation Versus Photon Involved-Field Radiotherapy for Patients With Solid Tumor Leptomeningeal Metastasis. J Clin Oncol 40 (33):3858–3867. 10.1200/JCO.22.01148
  - ○ This phase II clinical trial of proton CSI versus IFRT is the first to demonstrate a significant survival benefit from a radiation technique in the treatment of LMD.
- Glitza Oliva IC, Ferguson SD, Bassett R, Jr., Foster AP, John I, Hennegan TD, Rohlfs M, Richard J, Iqbal M, Dett T, Lacey C, Jackson N, Rodgers T, Phillips S, Duncan S, Haydu L, Lin R, Amaria RN, Wong MK, Diab A, Yee C, Patel SP, McQuade JL, Fischer GM, McCutcheon IE, O'Brien BJ, Tummala S, Debnam M, Guha-Thakurta N, Wargo JA, Carapeto FCL, Hudgens CW, Huse JT, Tetzlaff MT, Burton EM, Tawbi HA, Davies MA (2023) Concurrent intrathecal and intravenous nivolumab in leptomeningeal disease: phase 1 trial interim results. Nat Med 29 (4):898–905. 10.1038/s41591-022-02170-x
  - ○ The interim results from a phase I study of IV and IT nivolumab detail the emerging use of intrathecal immune checkpoint inhibition in the treatment of LMD.
- Kumthekar PU, Avram MJ, Lassman AB, Lin NU, Lee E, Grimm SA, Schwartz M, Bell Burdett KL, Lukas RV, Dixit K, Perron I, Zhang H, Gradishar WJ, Pentsova EI, Jeyapalan S, Groves MD, Melisko M, Raizer JJ (2023) A phase I/II study of intrathecal trastuzumab in human epidermal growth factor receptor 2-positive (HER2-positive) cancer with leptomeningeal metastases: Safety, efficacy, and cerebrospinal fluid pharmacokinetics. Neuro Oncol 25 (3):557–565. 10.1093/neuonc/noac195
  - ○ This phase I/II study of IT trastuzumab demonstrates the successful conversion of a non-CNS penetrant targeted therapy to an intraventricular formulation for the treatment of LMD.

## Data Availability

No datasets were generated or analysed during the current study.

## Declarations

### Statements and Declarations

This publication was supported by Grant Number UL1 TR002377 from the National Center for Advancing Translational Sciences (NCATS) to Ugur Sener.

This work was supported by National Institute of Health/National Cancer Institute P30 CA008748 and 1R01CA245499-01A1 to Adrienne A. Boire.
